# How do we measure gender discrimination? Proposing a construct of gender discrimination through a systematic scoping review

**DOI:** 10.1186/s12939-021-01581-5

**Published:** 2022-01-03

**Authors:** Laura de la Torre-Pérez, Alba Oliver-Parra, Xavier Torres, Maria Jesús Bertran

**Affiliations:** 1 Preventive Medicine and Epidemiology Department, Hospital Clínic de Barcelona, C/ Villarroel 170, 08036 Barcelona, Spain; 2 Consorci Sanitari de Barcelona, Carrer d’Esteve Terradas, 08023 Barcelona, Spain; 3 Clinical Health Psychology Section of the Institute Clinic of Neuroscience, Hospital Clínic de Barcelona, C/ Villarroel 170, 08036 Barcelona, Spain

**Keywords:** Sexism, Self-report, Systematic review [Publication type], Social discrimination, Mental Health

## Abstract

**Background:**

Gender discrimination (GD) has been frequently linked to mental health. The heterogeneity of how GD is defined has led to variation around the analysis of GD. This might affect the study of the association between GD and health outcomes. The main goal of this systematic scoping review is to operationalize the definition of the GD construct.

**Methods:**

Three search strategies were set in Pubmed, CINAHL and PsycINFO. The first strategy obtained results mainly about women, while the second focused on men. The third strategy focused on the identification of GD questionnaires. The prevalence of GD, factors and consequences associated with GD perception, and forms of discrimination were the principal variables collected. Risk of bias was assessed (PROSPERO:CRD42019120719).

**Results:**

Of the 925 studies obtained, 84 were finally included. 60 GD questionnaires were identified. GD prevalence varied between 3.4 and 67 %. Female gender and a younger age were the factors most frequently related to GD. Poorer mental health was the most frequent consequence. Two components of the GD construct were identified: undervaluation (different recognition, opportunities in access, evaluation standards and expectations) and different treatment (verbal abuse and behaviour).

**Conclusions:**

Two-component GD definition can add order and precision to the measurement, increase response rates and reported GD.

**Supplementary Information:**

The online version contains supplementary material available at 10.1186/s12939-021-01581-5.

## Introduction

Gender discrimination (GD) is a form of discrimination based on perceptions of gender and it has been evaluated through different perspectives. Overall, discrimination is considered to influence health. Thus, the heterogeneity in the analysis of GD can affect the study of its health consequences [1–4]. Reported health consequences have been mainly related to mental health, for example, occupational stress [5], feelings of vulnerability, and discomfort in women [4]. Other authors have found an association between discrimination and fatigue, depression, anxiety, rage, and alienation [6, 7].

Differences in GD analysis rely on the different approaches used. Questionnaires that measure reported GD [2] leave the responsibility for knowing what discrimination is to the subject filling in the questionnaire, and an individual’s concept of discrimination might vary in different cultural contexts. The other main approach is based on querying situations that are thought of as discriminatory [8, 9]. The forms of discrimination addressed differ between studies and usually lacked exhaustive characterization. Few studies clarify whether GD is referred to the experience of being discriminated against because of one’s gender, or the holding of attitudes/prejudices indicative of gender bias, commonly mixing these dimensions or not specifying them. The disparity leads to difficulties in the comparison of results. Moreover, the correspondence between these two approaches has not been evaluated.

This situation not only reflects the possibility of measurement of potentially different concepts under the umbrella of GD, but also a lack of consensus as to what GD operatively means. To our knowledge, unlike other related concepts, such as racial discrimination [10, 11], GD has not been operatively characterized from a behavioral and a psychological point of view.

Previous conceptualizations of sexism [12] accounted for discriminatory attitudes mostly towards women, despite gender being a key aspect in men’s health [13–16]. However, the nature of gender is considered to be performative rather than essential or possessive [17], meaning that it depends more on individuals’ attitudes or actions than on their sex. Thus, GD can be defined as a gender role prejudice directed towards men and women. Conversely, the intricate connections between the concept of sex and gender and different theorizations of the latter can explain why GD is mostly studied in women. Moreover, as socio-cultural contexts shape gender roles, the analysis of its influence is a cornerstone in GD’s conceptualization and interpretation [18]. This adds complexity to the definition of GD and has led to different conceptions of GD across the literature.

Constructs are defined based on different methodologies (see [19]) and its validation is complex, usually based on the analysis of its association with external factors. Scoping reviews are normally used to clarify definitions and the conceptual boundaries of a topic [20]. Through this methodology, it is possible to compile information that could be useful for external validations of the construct. Moreover, systematic scoping reviews facilitate understanding of the research processes undertaken to achieve the conclusions of a particular study. Therefore, this methodology can be used to summarize the concept of GD and clarify its operative definition while assessing studies’ risk of bias, and identifying future lines of research [21].

To develop an operative GD construct could contribute to increasing the homogeneity of the GD and the analysis of its different dimensions as health determinants. Furthermore, it could also help to understand how GD association with health works and which dimensions have more impact on health.

In order to frame how GD is currently being studied and its association to health, this systematic scoping review aims to identify GD questionnaires and their psychometric properties and, with this information, to propose the dimensions of the GD construct. Moreover, to contextualize the proposal and facilitate future external validations of the GD construct, a secondary objective is to collect the associated factors related to the perception of discrimination and to describe the mental health consequences associated with experiences of GD.

## Materials and methods

This systematic review has been registered at the International Prospective Register of Systematic Reviews (PROSPERO, https://www.crd.york.ac.uk/prospero/) with the registration number CRD42019120719.

### Search strategy

The online databases Pubmed, CINAHL, and PsycINFO were included. The initial search was performed in May 2018 and was updated in April 2019.

The search strategy (SS) was performed using the following terms: “Sexism”, “Gender discriminat*”, “Social perception”, “Self-concept”, “Hypermasculinity”, “Masculinity”, “Gender Identity”, “Survey*”, “Questionnaire*”, “Self-report*”, “Perception*” (See S1_File for the detailed search strategies). Although gender definition and gender-based power structures are out of the scope of this review, three different SS were defined to explore all potential aspects of GD. The first SS focused on the identification of the components of GD. It included general terms related to GD (“Sexism”[MeSH] and “Gender Discrimination”) and was narrowed (by the Boolean operator [AND]) with the terms “self-concept” and “social perception” to allow a number of results assumable by the research group. As the results with this SS were mainly related to GD towards women, the second SS was aimed at exploring different dimensions of GD towards men. The second SS included the terms “Hypermasculinity” and “Masculinity”, which are modified terms related to discrimination, and it was narrowed (by the Boolean operator [AND]) with the term “Gender Identity” [Mesh major topic] to find GD and gender role prejudice in relation to men. The third SS was defined to identify discrimination scales. The scales reflect what is understood as discrimination on different axes (gender, race, sexual orientation, or other), which are considered to be related [22]. It included general terms related to GD (“Sexism” and “Gender Discrimination”) in the title or abstract and “self-report*” and “perception*”. The SS was adapted for the CINAHL and PsycINFO databases. An additional file shows the different search strategies in more detail [see Additional file 1].

In order to ensure the collection of different approaches to GD, no specific study designs were excluded. Only adult populations were included and there was no limitation on the publication date. The publication state was defined as published and available online, and the language used could be Spanish, English, French, or Portuguese, which reflected the language background of the reviewers. Studies were also identified through bibliographic references.

### Analysis strategy

Two different analysis strategies (AS1 and AS2) with different inclusion criteria were defined. In the analysis strategy number 1 (AS1) all the defined variables were collected (variables in detail in the S1 and S2 Table [see Additional file 2]). On the one hand, the main study results were information on forms of discrimination and potential triggers of discrimination, in order to identify GD dimensions. On the other hand, information about factors associated with greater or lower GD perception and potential consequences of GD reporting were collected, to facilitate future GD construct validation. Additionally, other results such as the main study result or perceived discrimination prevalence were collected to contextualize the findings. Studies were included if their focus was either the analysis or the perception of discrimination. Also, studies analyzing the effects of discrimination and the factors associated with the perception of discrimination were included. The final inclusion in AS1 was linked to the presence of information of any of the result-study variables (Table S2 [see Additional file 2]). AS2 collected data about questionnaires on discrimination.

Articles not fitting AS1 inclusion criteria were included in the AS2 if their main focus was the analysis of the perception of discrimination. Final inclusion in AS2 was tied to the use of a questionnaire on discrimination. The exclusion criteria were the same for the two analysis plans. The exclusion criteria were (1) the inclusion of biological effects/measurements in the objectives of the study (i.e. hormonal levels, image tests); (2) the analysis of discriminatory practices, as they did not analyze GD perception; (3) descriptive analysis of the effects of gender role, as was out of the scope of the review; (4) measurement of health effects not related to mental health. As the effects of GD on physical health can be both direct and indirect, the measurement of health effects not related to mental health is conceptually more complex; (5) the descriptive analysis of GD time trends and (6) the analysis of experiences associated with cancer treatments, as the complexities of  gender and GD perceptions are out of the scope of this review.

The two screening phases (first by titles, second by abstracts) were performed independently by two reviewers. A conservative strategy was defined in which studies not naming discrimination itself were excluded from the review in order to avoid misinterpretations of the concept. The concordance in the screening and eligibility was registered and the reasons for exclusion were registered in a free-text field.

The process of gathering all data (variables in detail in the S1 and S2 Table [see Additional file 2]) was performed twice, by two reviewers independently. The process was overseen by the other two reviewers, who verified the optimal data collection procedure. The principal objective was prioritized - for the construct definition, the articles with information about forms of discrimination, triggers, and scale items listed were analyzed. The analysis consisted of inspecting data for shared properties of different forms of discrimination or triggers to define categories from which theoretical ideas could be generated. To allow this process data was broken down, compared and categorized using both inductive and reductive processing. Inductive reasoning refers to the process of making broad generalizations, that were afterwards grouped (or reduced) to similar themes, allowing the identification of categories. Identified categories defined the operative components of the construct. The process was completed through consensus of the different reviewers, considering categories frequency.

The risk of bias in individual studies was assessed only in the articles where forms of discrimination and triggers were collected to estimate the methodological adequacy of the articles included in the construct proposal, as this was the main goal of the scoping review. It was performed with different scales depending on the study design using Joanna Briggs Institute Critical Appraisal Tools [23]. In the case of mixed studies, the “Assessment of the mixed methods design of studies in HSR” tool was used [24].

## Results

### Systematic review process

**Fig. 1:**
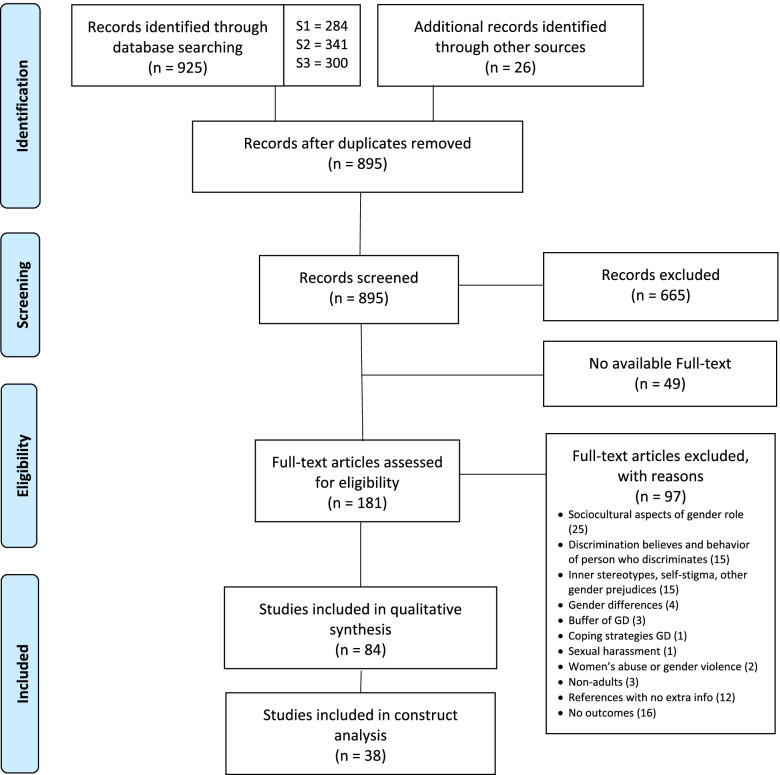
Flow Diagram. SS1, Search Strategy (1) SS2, Search Strategy (2) SS3, Search Strategy (3) GD, Gender discrimination

A total of 925 studies were included in the review through the search strategies used. Fifty-six duplicates were identified. Additionally, 26 references were included in the analysis. Six hundred and sixty-five references were excluded during the screening. One hundred and eighty-one articles were available in full text to the research group. Of those, a total of 97 articles were excluded from the review; exclusion reasons are shown in Fig. 1. A total of 84 papers were included with more than 96 % agreement among reviewers, 71 from the AS1 and 13 from AS2.

#### Study characteristics

General characteristics of the studies included are available in S3 Table (Additional file 2). Forty of the 71 studies (AS1) were exclusively focused on GD and 17 on gender-based discrimination in addition to other discrimination axes (57 GD related articles). Among those studies which focused only on GD, most of them (n=29) assessed discrimination in relation to women. S4 Table shows other study characteristics in more detail [see Additional file 2].

Thirty-eight of the 71 studies (AS1) described different forms of discrimination - these studies were addressing either GD (n=27), GD and other discriminations (n=3), or other discriminations only (n=8). The settings were mainly work-related (n=23) or on a daily basis (n=8). No triggers in GD studies were described, and there were only three studies where these could be identified [25–27].

### Gender discrimination questionnaires

Of the 57 studies addressing GD in the AS1, 47 used at least one questionnaire to assess discrimination. The review identified a total of 75 studies using questionnaires, 62 by the AS1 and 13 through the AS2. Thirty-five surveys addressed only GD, 24 GD and multiple axes, and 16 other kinds of discrimination.

Less than half of studies assessing GD (n=23) used a questionnaire customized by the research group, among them, internal consistency was assessed in 12, and one study of these performed a factor analysis [28]. The rest of the studies (n=36) used already constructed questionnaires, Krieger et al. [29] being the most used (n=8), followed by Schedule of Sexist Events [30] (n=4). From the 59 GD studies (47, AS1 and 12, AS2), 26 addressed the declaration of the perception of discrimination with a Yes/No question, whereas 32 asked for specific forms of what was considered discrimination, and one study did not provide data about the questionnaire’s items.

### Approaches to discrimination

Most approaches in GD analysis differed only in the kind of questionnaire used. However, two relevant approaches varied between studies: the differentiation between observed and experienced discrimination and the conceptual framework used.

#### The differentiation between observed and experienced discrimination

Perception of discrimination was inconsistently assessed in observers and victims. Five studies addressed this difference by asking specifically about this aspect [31–35]. However, of the 20 studies assessing gender group discrimination, ten did it in mixed populations: five asked for perceived GD towards their in-group (women were asked about GD towards women and men towards men), four studies asked for perceived gender bias only towards women and one had an unclear approach.

#### Conceptual frameworks

Some of the included studies had different comprehensions of GD and gender-prejudice related concepts. The distinction between GD and other forms of gender-based prejudice is referred to by Berg [37]; Hang-yue et al. [38] and Nye et al. [36]. Through a confirmatory factor analysis, Nye et al. [36] refer to sexist behavior as a different concept from GD. Sexist behavior would be considered a form of hostile sexism and part of a broad hostile sexism construct but separated from GD. Sexist behavior would include those acts that are clearly gender-related and are usually linked to colleagues or peers, whereas GD would be inherently less explicit and refer to motivations behind behaviors. Other studies allude to “gender-related stressors” [37] and “gender bias” [38] as different forms of gender-based prejudice. Berg [37] used “gender stressors” to refer to the stress related to the cultural expectations and role burden of women (thus, related to the gender role). Meanwhile, gender bias was considered by Hang-yue et al. [38] as discrimination towards a group of women within the workplace or society, defining GD as an individual experience. Moreover, some researchers consider diverse situations or events (such as, sexual objectification and sexual harassment) as GD [37, 39–41] while the same group of situations is thought to be part of different constructs or dimensions of gender-related prejudice by others [36].

Frameworks also refer to how GD is interpreted, contextualized and how it relates to other variables. Gomez [42], states that cross-group discrimination implies inferiorizing and thus, the group used an inferiorization scale to assess discrimination. Reid [40] introduced the discrepancy fact measure scale to assess the tendency to minimize or maximize measurable information from the social context about GD as a possible variable affecting GD perception. Also, recently, Kira et al. proposed a trauma-based framework for GD [43] developed on different subscales that comprised perceptions of, and attitudes towards women in a familiar environment.

### Study outcomes

#### GD perception

Only one study [37] reported that all women in the sample had perceived some kind of GD throughout their lives. Perceived discrimination among the rest of the studies assessing GD (n=56/57 in AS1) was between 3.4 % and 67 %. Thirty-two studies assessing GD reported associated factors with it. Female gender was the most frequent factor associated with GD in mixed populations (n=7). S5 Table shows other associated factors in more detail [see Additional file 2].

#### Variables affecting GD perception

Fewer studies focused on how different variables could affect the perception of GD. Seven studies [33–35, 41, 44–46] contemplated the role of the person who discriminates in the perception of discrimination. Flippen and Parrado [47] addressed the relevance of the kind and origin of the discriminatory behavior, finding that the relationship between the person who discriminates and the kind of behavior, modifies the perception of GD. In this study, more women reported discrimination when it was through comments made by their boss or a stranger in comparison to their boyfriend. Another example is the study of the interactions between different axes of discrimination. Harnois [48] studied the relationship between age, gender, and ethnic-based discrimination and found that reporting discrimination on one axis of inequality was also a risk factor for the perception of discrimination on others [48].

Ten studies reported factors associated with lower GD perception. Three studies reported different ethnicities as a factor (Caucasic, Black, Latin, or Asian) [49–51]. Other studies referred to patriarchal society [8, 52], beliefs about gender roles, conservative political ideas, working in basic science or as staff in academic environments, age (older) and gender (men), and gender expression among women (more masculine women tended to perceive less discrimination). Hinze et al. [53] reported that when exposed to sexual harassment, less personal forms of harassment were associated with lower discrimination perception. Also, the group addressed that fears of being sensitive or rejecting victimhood positions were also associated with lower GD perception [53].

#### Associations with GD

Twenty-one of 57 studies reported possible effects associated with GD, and 17 of these articles only assessed GD among women. They could be divided into either personal or professional. Among men and women, personal consequences were mostly related to health (consequences can be seen in detail in the S6 Table [see Additional file 2]**)**. The most frequent was poor mental health, and it was measured with a validated scale of anxiety or depression in four of the studies[2, 3, 54, 55].

Regarding studies of other axes of discrimination (n=14), in approaches used, one study measured sexual identity discrimination [25] as two dimensions; environmental and interpersonal microaggressions. As in the study of GD, associated factors with reporting were younger age, gender female [56, 57] and higher education [47], but these were not consistent in all of the studies. Meanwhile, all the studies reporting the possible consequences of GD (n=7) [25, 26, 35, 58–61] described higher psychological distress in those who perceived discrimination.

### Gender discrimination as a construct

**Fig. 2:**
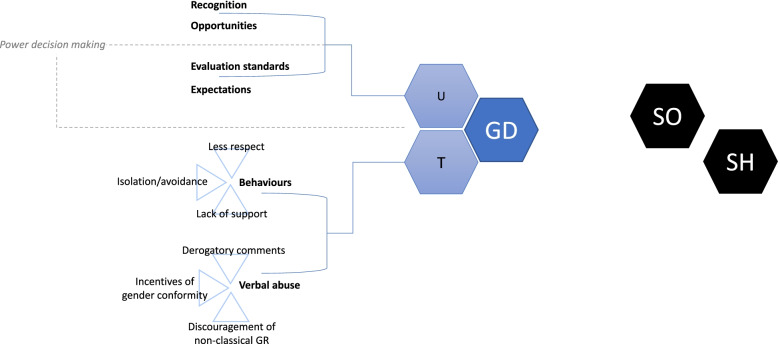
Gender Discrimination construct map. GD: Gender Discrimination U: Undervaluation. T: Different Treatment. SO: Sexual Objectification. SH: Sexual Harassment. GR: Gender Role

Two major dimensions of the construct of GD were identified: undervaluation (1) and different treatment (2). The graphic representation can be seen in Fig. 2.

First, *Undervaluation* refers to the expressed lower value of a person’s capacity as a consequence of their gender. It is a subtle act over actions or performance of a person and includes four subdimensions: differences in recognition, in opportunities, in evaluation standards and in expectations. Different recognition refers to the attribution of varying merit to similar performed activities (such as different salaries), whereas opportunities are referred to the differences in access to higher social status or to resources (such as being hired for specific jobs; access to scholarships, or other resources). Differences in evaluation standards refer to biased evaluation based on gender roles; for example, to assume that some skills might not have been acquired, because of the gender role. Different expectations encompass expected attitudes, skills, or levels of proficiency in gender role segregated areas. For example, to expect that a woman will not understand a car engine or that a man will be uncomfortable around displays of emotions. This would also include work-related expectations that could determine work assignments.

Second, *Different Treatment* is related to evident and obvious actions towards a person due to their gender. This dimension is manifested in a more direct way than *Undervaluation.* It includes *Different Behavior* towards a person (less respect, more isolation and lack of support) and verbal abuse or comments directed towards people because of their gender. Comments could be divided into different groups: derogatory comments towards one’s gender (including offensive jokes), incentives of gender conformity and discouragement of non-classical gender role activities, behaviors or attitudes.

Lack of decision-making power was an aspect identified through two studies in women. It would reflect the lack of independence in the family environment as a form of disadvantage experienced by women. It could be part of the dimension of *Undervaluation* or a dimension by itself (see Fig. 2). However, no saturation with other studies was identified and this was not finally included in the construct proposed.

Sexual objectification and sexual harassment were identified in questionnaires and interviews, but these aspects were not included in the construct proposal. Data included in non-GD studies were used only to support identified dimensions. Data related to reactions to discrimination, self-stigma, or gender violence were excluded from the construct. S7 table in an additional file shows the studies linked with the main dimensions identified [see Additional file 2].

### Risk of bias assessment

The risk of bias was assessed in the 33 studies that reported either forms or triggers of discrimination. No specific scale was identified to assess biases for validation studies (n=4) and literature review (n=1). The risk of bias assessment can be seen in S8 Table [see Additional file 2].

## Discussion

GD is measured in a wide variety of contexts and with several methodologies. Different measures regarding the target audience (men, women or both), and the type of discrimination assessed (individual, group) hindered comparison of the results.

The different scales used, and the various study contexts (settings and populations) could explain the wide range of declared discrimination and the plethora of factors associated with it. Moreover, conceptual frameworks changed across the studies. Some research groups clearly state different concepts of gender prejudice [36–38] and diversely define what GD is. Meanwhile, others include GD concepts such as sexual harassment or sexual objectification [37, 39–41], which have been studied separately from GD [62, 63]. Also, some studies approached the perception of GD in the same way as observed GD, which may be different constructs or dimensions.

Further heterogeneity can be observed in the variety of factors associated with reported GD. The factors associated with GD could indicate that either in some settings, gender discriminatory attitudes are more present than in others or that specific contexts or characteristics can increase the perception of GD. However these differences are not clarified in the studies identified through this review. The generalization of this data could be misleading if the social context is not considered. More in-depth mixed-method studies comparing settings and social contexts could help understand these nuances. Besides, few studies in the review contemplated the importance of interactions and intersectionality in their study design. As it was not the focus of the review, this could be explained by the search strategy used. However, the effect of other discriminations should always be taken into account [48, 64].

More homogeneity has been found in the consequences of discrimination, and the differences identified may be more related to the measuring tools. Psychological distress and lower self-concept seem to appear consistently due to either GD or other types of discrimination. Outside the focus of this review, some studies [37, 65] identified factors that would modify discrimination effects which could be useful to approaching GD as a determinant of health.

GD was summarized in two dimensions; *Undervaluation* (1) and *Different Treatment* (2). Both dimensions were present in most of the studies assessed. *Undervaluation* is a more subtle aspect of GD whereas *Different Treatment* refers to evident actions. In one study [8], the lack of decision-making power or independence in the family environment is identified as a form of disadvantage among women and a way of undermining female capacities. The lack of decision-making power was also introduced by Kira et al. [43] through several items in the Gender Discrimination Inventory, also referring to the familiar environment. This aspect could be extrapolated to a lack of decision-making power in a specific role setting (which could be different from culture to culture depending on gender roles). So, conceptually, it could be either included in the *Undervaluation* dimension or considered as an entity in its own right. However, due to the lack of reiteration of this possible factor, it could not be included in the construct proposed.

The different frameworks identified were considered in the analysis of questionnaire items for the construct proposal. Forms of activity such as sexist behavior (as described by Nye et al. [36]) or gender bias (as defined by Hang-yue et al. [38]) were considered to be GD as they were in other studies. However, as sexual objectification has been studied separately [62, 63], and sexual harassment and gender violence have legal implications, these elements were identified but separated from the analysis.

The proposed construct presents a clear distinction between subtle and explicit ways of discrimination. The first dimension, *Undervaluation*, even if it can be systematically present in institutions, can be both culturally invisible and difficult to objectify. Whereas *Different Treatment*, especially in forms of verbal abuse, is more explicit and can be noticed by both the person to whom it is directed and by other external observers. So, even if the experience of discrimination is highly subjective and recall bias might influence responses, some GD forms could be more easily reported even in settings where GD is culturally embodied. On the other hand, perceptions of more subtle forms of discrimination, as *Undervaluation* subdimensions, specify mediums through which embedded GD can occur. The recognition in the construct of these specific forms of submerged GD make them more tangible and can help overcome biased perceptions and promote individual agency over the influence of social contexts in a person’s core beliefs.

Previous GD definitions identified in the review are mostly related to the work environment [36], and others were developed grounded on the assumption that GD prejudice could only be directed towards women [38, 43]. The proposed construct could be applicable in every setting because it is based on studies developed in different environments with a representation of public settings and personal relationships. Also, it is thought to be directed towards both genders. However, given the few studies included addressing GD towards men, there is little evidence supporting the applicability of this construct to both genders, and further research is needed to confirm this hypothesis.

Fewer studies addressed discrimination in gender-mixed populations, reporting gender differences in perceived discrimination. However, most of these studies assessed perceived discrimination with a Yes/No question of declared discrimination. This approach implies that men can easily identify gender prejudice in different contexts, which would seem unlikely given the differences in gender roles and privileges.

During the screening of relevant studies for this review, studies dealing with the deleterious effects of masculinities by focusing on the study of self-worth and self-stigma [27, 66] were identified but not included. Others addressed masculinity threats [67, 68] or approached gender prejudice by focusing on anti-effeminacy attitudes [27, 69]. These attitudes are also present in homosexual self-discrimination [27, 58, 70] and as a determinant of homophobic attitudes [71, 72]. All these directly or indirectly refer to how men limit their behavior and value their self-worth according to how they fit their gender role.

Given these lines of research, it is possible that GD in men may involve a specific dimension of self-stigma that modifies how men perceive GD. Men would control their behavior in order to be less feminine and value themselves according to the level of control [67]. This dimension would manifest not only when they are in situations outside their gender role, but also for the fact of being men. The lack of help-seeking [58], or the absence of the display of emotion in men [73] could not only be related to the role, but also to the self-stigma [58]. However, multiple concepts related to this possible dimension can be identified and further studies are needed to explain and characterize how these multiple concepts can be encompassed and if these dimensions exist or could relate to gender stressors in women. These forms of gender-based prejudice might not only modify perceptions of discrimination but also determine the definition of further dimensions of GD. A deeper analysis of the proposed dimensions, considering gender conceptions, the relationship between gender, sex and power structures is needed to further clarify the construct.

When assessing the risk of bias, the studies included followed an adequate methodology. However, it is worth mentioning that the most crucial part of the studies’ potential biases, which are the characteristics of the measurement tools used, could not be fairly assessed. The development of a specific assessment scale could be useful in addressing this gap in the future.

There are several aspects of this study that may limit the generalizability of the results. First, given the analyzed data, the proposed construct might be over-influenced by North American culture. Thus, it should be interpreted carefully in different social contexts and settings, as aspects like GD perception, factors associated with GD perceptions or GD forms themselves might manifest in different ways. Additionally, interactions between forms of discrimination, self-stigma dimensions, and effect modifiers of perceived discrimination were not assessed in the conceptual framework of the review, and they might play a key role in GD and its relationship to health. Elements such as gender identity or past experience could play a determinant role in GD perception, and these were not considered in the proposed construct. Second, regarding the methodology, only published articles were included, so there is a possible publication bias in the results of this review [74]. Besides, bias assessment lacks qualitative evaluation, which is usually recommended. Third, the results regarding reported discrimination and associated factors are based on the results of different questionnaires, which have different items, scale punctuation and interpretations, so this limits the comparability of the results. Moreover, collected data for external validation was limited (for example, only collecting associations with mental health outcomes) and further data collection on different factors associated with GD should be performed to fully contextualize the GD construct validation. Finally, the narrowed search strategy may have led to the missing of relevant studies in GD. However, given the amount of studies focused on GD and related issues and the technical complexity of the review, the use of a limited and feasible search strategy was the cornerstone for making this study possible. Also, the defined search limited by Mesh terms and key concepts has led to the inclusion of recent research.

Even with the mentioned limitations, this systematic scoping review offers a rigorous methodology to deepen the understanding of GD, how its perception can be measured and the importance of properly identifying the factors that can be leading to a biased perception of GD within the methods used to measure it. Even in different cultural contexts, the homogeneity of a definition could be a helpful tool for later social-context based interpretations. Moreover, the methodology facilitates the identification of gaps of information or future research lines [20].

## Conclusions

The construct proposal gathers the different forms of GD identified in the studies included in this review. Different GD measurement tools can be validated for measuring one or more dimensions, adding precision to the questionnaires used. Moreover, the specification of which dimensions of GD are measured, can help to understand GD prevalence results. As to how questions are asked can influence a subject’s responses [75], this construct may increase response rates and refine the perceptions of discrimination. By identifying the two main dimensions and subdimensions whereby GD may be held, a conceptual map is outlined to help navigate GD research through social contexts and individual core beliefs. As such, GD construct can be a tool to help identify more specific approaches to study GD, going beyond asking about reported GD (by Yes/No response) and avoiding recall bias in those who did not experience GD events as traumatic. This deeper insight into how GD takes place, gives a more comprehensive approach to its presence in society and its repercussions on individuals, and possibly, its role as a determinant of health. Nonetheless, further studies should be performed in order to validate and put this construct into practice.

## Supplementary Information

**Additional file 1. **Search strategies.

**Additional file 2. **Supporting information.

## Data Availability

Qualitative data collected regarding abstract screening and variables collection was made through Microsoft Access and exported in excel sheets.
