# Supplementary material for: How do we measure gender discrimination? Proposing a construct of gender discrimination through a systematic scoping review
Source: Int J Equity Health. 2022 Jan 3;21:1. doi: 10.1186/s12939-021-01581-5 (PMC8722302; doi:10.1186/s12939-021-01581-5)
Supplement: Supplementary file 1 — Additional file 1. Search strategies. [file 12939_2021_1581_MOESM1_ESM.docx]

**Additional File 1. Search Strategies**

In this file the search strategies are detailed.

**Pubmed search strategies**

Search strategy 1

(((sexism[MeSH Major Topic]) OR Gender Discrimination[Title/Abstract])) AND ((social perception[MeSH Terms]) OR self concept [MeSH Terms])

*Key concepts of the review*

1: Sexism [MeSH Major Topic]

2: Gender Discrimination [Title/Abstract]

3: 1 or 2

*As the strategy was too broad and due to the complexity of the strategy, the search was narrowed with:*

4: Social Perception[MeSH Terms]

5: Self Concept [MeSH Terms]

6: 4 or 5

*We narrowed the search using the Boolean operator [AND]*

7: 3 and 6

Filter: published articles*

Search strategy 2

*This strategy aimed to find studies that focused on how men could be discriminated just because of being men in order broaden the classic scope of gender discrimination, as the first search strategy only found studies on gender discrimination against women.*

((((((Hypermasculinity[Title]) OR ""Masculinity""[ MeSH Major Topic]) AND Gender Identity*[MeSH Major Topic])) AND (((""Self Concept""[ MeSH Terms]) OR ""Social Perception""[ MeSH Terms] OR ""Discrimination""[MeSH Terms]))))

1: Hypermasculinity[Title]

2: Masculinity [MeSH Major Topic]

3: 1 or 2

*Search in the point 3 found too many studies not related with main focus of the review, so, the search was narrowed with the step 4.*

4: Gender Identity*[MeSH Major Topic]

5: 3 and 4

*We still found too many unrelated topics and narrowed the strategy by concepts related to discrimination.*

6: Self Concept [MeSH Terms]

7: Social Perception [MeSH Terms]

8: Discrimination [MeSH Terms]

9: 6 or 7 or 8

*Finally, the search was narrowed with the Boolean operator [AND]*

10: 5 and 9

Filter: published articles*

Search strategy 3

*This strategy aimed to find questionnaires in gender discrimination and others. MESH terms were prioritized instead of other terms to allow a broader scope which led to identifying other studies that were not detected with the rest of the strategies.*

(((((((((Gender Discrimination[Title/Abstract]) OR Sexism[Title/Abstract])) AND ((Self-report*[Title/Abstract]) OR Perception*[Title/Abstract])

1: Gender Discrimination[Title/Abstract]

2: Sexism[Title/Abstract]

3: 1 or 2

*To find questionnaires or surveys the search was narrowed with the terms:*

4: Self-report*[Title/Abstract]

5: Perception*[Title/Abstract]

6: 4 or 5

7: 3 and 6

Filter: published articles*

**CINAHL search strategies**

*Interface: EBSCOhost Research Databases. CINAHL complete Database.*

Search strategy 1

*Key concepts of the review.*

1: Sexism

2: Gender discrimination

*The following expression was added to broaden the view due to the more flexible style titles and expressions of qualitative studies included in CINAHL database.*

3: Social Discrimination/psychology*

4: 1 or 2 or 3

*As the strategy was to broad and due to the complexity of the strategy, the search was narrowed with:*

5: Social Perception

6: Self Concept

7: 5 or 6

*We narrowed the search using the Boolean operator [AND]*

8: 4 and 6

Filter: published articles* Full text available*

Search strategy 2

1: Hypermasculinity

2: Masculinity

3: 1 or 2

*Gender identity narrowed the strategy too much in this database, so we decided to broaden the perspective in this case and did not include “Gender identity” as a search term.*

4: Self Concept

5: Social Perception

6: 4 or 5

*Finally, the search was narrowed with the Boolean operator [AND]*

7: 3 and 6

Filter: published articles* Full text available*

Search strategy 3

1: Gender Discrimination [Title/Abstract]

2: Sexism[Title/Abstract]

3: 1 or 2

4: Self-report[Title/Abstract]

5: Perception[Title/Abstract]

6: 4 or 5

*Narrowing using the Boolean operator [AND]*

7: 3 and 6

Filter: published articles* Full text available*

**PsycINFO search strategies**

Interface: APAPsycNET. Database: PsycINFO.

Search strategy 1

((((MeSH: (self concept)))) OR (((MeSH: (social perception))))) AND ((((abstract: (gender

discrimination)))) OR (((MeSH: (sexism)))))

*Key concepts of the review. As PsycINFO accepted MESH terms, they were included.*

1: Sexism [MeSH]

2: Gender Discrimination [abstract]

3: 1 or 2

4: Social Perception [MeSH Terms]

5: Self Concept [MeSH Terms]

6: 4 or 5

*Narrowing using the Boolean operator [AND]*

7: 3 and 6

Filter: published articles*

Search strategy 2

((((MeSH: (discrimination)))) OR (((MeSH: (social perception)))) OR (((MeSH: (self concept))))) AND

((((abstract: (hypermasculinity)))) OR (((MeSH: (masculinity)))))

1: Hypermasculinity [abstract]

2: Masculinity [MeSH]

3: 1 or 2

*As for the CINAHL strategy, Gender identity narrowed the strategy too much in this database, so we decided to broaden the perspective in this case and did not include “Gender identity” as a search term. Moreover, the step 6 was added to broaden the search (less results were obtained and thus it was possible to broaden it).*

4: Social perception [Mesh]

5: Self Concept [Mesh]

6: Discrimination [Mesh]

7: 4 or 5 or 6

*Finally, the search was narrowed with the Boolean operator [AND]*

8: 3 and 7

Filter: published articles*

Search strategy 3

((((abstract: (gender discrimination)))) OR (((abstract: (sexism))))) AND ((abstract: (perception*)))

AND ((((abstract: (survey*)))) OR (((abstract: (questionnaire*)))))

1: Gender Discrimination [abstract]

2: Sexism [abstract]

3: 1 or 2

4: Perception[abstract]

*After testing it, we observed that the term self-report narrowed the strategy too much (1) and did not obtain questionnaires (2). Keeping the term perception (which made the strategy too broad), we added the terms “survey” and “questionnaire” in order to provide the maximum questionnaires possible.*

5: Survey [abstract]

6: Questionnaires [abstract]

7: 5 or 6

8: 3 and 4 and 7

Filter: published articles*

**Limits on language and population were applied during the studies’ first screening.*

- *Language: English, French, Portuguese, Spanish.*
- *Adult populations*
