# Supplementary material for: How do we measure gender discrimination? Proposing a construct of gender discrimination through a systematic scoping review
Source: Int J Equity Health. 2022 Jan 3;21:1. doi: 10.1186/s12939-021-01581-5 (PMC8722302; doi:10.1186/s12939-021-01581-5)
Supplement: Supplementary file 2 — Additional file 2. Supporting information. [file 12939_2021_1581_MOESM2_ESM.docx]

**Additional File 2. Supporting information.**

Additional information of the scoping review results can be found in the following tables:

| \| S1 Table. Variables of study characteristics \| 2 \| \| --- \| --- \| \| S2 Table. Variables of study results. \| 2 \| \| S3 Table. Included studies characteristics. \| 3 \| \| S4 Table. GD study characteristics \| 3 \| \| S5 Table. Associated factors to increased perception of GD \| 4 \| \| S6 Table. Reported associations to perception of discrimination \| 5 \| \| S7 Table. Studies used to induce the construct. \| 6 \| \| S8 Table. Risk of Bias of studies used for construct definition. \| 7 \| |  |
| --- | --- | --- | --- | --- | --- | --- | --- | --- | --- | --- | --- | --- | --- | --- | --- | --- | --- |
|  |  |
|  |  |
|  |  |
| **S1 Table. Variables of study characteristics**   \|  \|  \| **Variable** \| **Categories/description** \| \| \| --- \| --- \| --- \| --- \| --- \| \| **Study characteristics** \|  \| Title \|  \| \| \| Publication year \|  \| \| \| First author \| When only two authors are listed, the two authors were included in this variable \| \| \| Design \| Cross-sectional  Case-control  Cohorts  Qualitative \| Mixed-method  Systematic Review/metanalysis  Quasiexperimental  Other \| \| Study location \| Location where the study was developed. \| \| \| Sample size \| Number of subjects included in the study \| \| \| Analysis unit \| Individual/population/both individual and population \| \| \| **Population characteristics** \| Mean age \| In those reporting mean age \| \| \| Gender \| Women  Men  Both genders \| \| \| Ethnicity \| Only Caucasic  Only Asian/Asian origins  Only African/African origins  Only middle east/middle east origins \| Diverse: homogeneous distribution  Diverse: non-homogeneous distribution  Not disclosed \| \| Social class \| Vulnerability situation  Non-vulnerability situation  Not disclosed \| \| \| Sexual orientation \| Homosexual  Bisexual  Heterosexual  Others  Not disclosed \| \| \| Discrimination axes \| Gender  Race  Social class \| Age  Sexual orientation or identity  More than one of the above \| \|  \| Discrimination perception type \| Individual / Group / Both individual and group \| \| \| Discrimination setting \| Work  University education  Working internship  Healthcare \| Family environment  Outside family environment  Daily basis  More than one of the above \| \| Scale/measurement of discrimination \| Yes  No \| \| |  |
|  |  |
|  |  |

**S2 Table. Variables of study results.**

|  | **Variable** | | **Categories/description** |
| --- | --- | --- | --- |
| **Study results** | Main result | | Most outlined result in the study |
|  | Situations/actions that trigger discrimination | | Situations identified in the study as triggers of discrimination |
|  | Forms of discrimination | | Actions or situations included in the studies as discrimination |
|  | Perceived discrimination prevalence | | Percentage of individuals that perceived discrimination |
|  | Associated factors to GD perception | Risk factors | Factors that associated greater discrimination perception |
|  |  | Protective factors | Factors that associated lower discrimination perception |
|  | Discrimination consequences | | Factors associated with the perception of discrimination. Either these factors are reported by the study subjects or are measured with scales (i.e. self-esteem scale and discrimination perception). In qualitative studies the experiences after discrimination perception were collected. |
|  | Anxiety or depression was measured in the study with a validated scale | | Yes/No. Questionnaire validation was considered as so when the authors refer at least a parameter of internal consistency (i.e. Cronbach alfa). Only anxiety or depression measurements were considered as Yes. If the study relates this anxiety or depression to perceived discrimination. |

GD, Gender discrimination

| **Characteristic** | **N** | **%** |
| --- | --- | --- |
| **Search strategy*** |  |  |
| 1 | 39 | 46.43 |
| 2 | 5 | 5.95 |
| 3 | 40 | 47.62 |
| **Analysis strategy*** |  |  |
| 1 | 71 | 84.52 |
| 2 | 13 | 15.48 |
| **Publication year*** |  |  |
| Before 2000 | 4 | 4.76 |
| 2000-2010 | 26 | 30.95 |
| After 2010 | 54 | 64.29 |
| **Discrimination axis**** |  |  |
| Gender | 40 | 56.34 |
| Race | 6 | 8.45 |
| Sexual orientation | 5 | 7.04 |
| Social class | 1 | 1.41 |
| Religion | 1 | 1.41 |
| More than one | 18 | 25.35 |
| **Perception**** |  |  |
| Individual | 50 | 70.42 |
| Group | 2 | 2.82 |
| Both | 19 | 26.76 |
| **Setting**** |  |  |
| Professional (work. university. work training) | 37 | 52.11 |
| Daily basis (all settings) | 15 | 21.13 |
| Public settings | 13 | 18.31 |
| Close relationships | 3 | 4.23 |
| **Study design**** |  |  |
| Cross-sectional | 53 | 74.65 |
| Qualitative | 4 | 5.63 |
| Mix-method | 3 | 4.23 |
| Other | 11 | 15.49 |
| **N**** |  |  |
| 0-100 | 11 | 15.49 |
| 100-500 | 21 | 29.58 |
| 500-1000 | 12 | 16.90 |
| >1000 | 26 | 36.62 |
| **Origin**** |  |  |
| North America | 46 | 64.79 |
| Asia | 8 | 11.27 |
| Europe | 9 | 12.68 |
| Africa | 4 | 5.63 |
| Oceania | 2 | 2.82 |
| **Mean age**** |  |  |
| 18-30 | 14 | 19.72 |
| 30-45 | 11 | 15.49 |
| 45-60 | 3 | 4.23 |
| >60 | 1 | 1.41 |

**S3 Table. Included studies characteristics.**

GD studies N=57.†studies only focused in GD N=40

**S4 Table. GD study characteristics**

| **Characteristic** | **N** | **%** |
| --- | --- | --- |
| **Publication year** |  |  |
| Before 2000 | 3 | 5.26 |
| 2000-2010 | 20 | 35.09 |
| After 2010 | 34 | 59.65 |
| **Perception** |  |  |
| Individual | 37 | 64.91 |
| Group | 2† | 5.00 |
| Both | 18 | 31.58 |
| **Setting** |  |  |
| Professional (work. university. work training) | 32 | 56.14 |
| Daily basis (all settings) | 12 | 21.05 |
| Public settings | 7 | 12.28 |
| Close relationships | 3 | 5.26 |
| Healthcare | 2 | 3.45 |
| **Study design** |  |  |
| Cross-sectional | 40 | 70.18 |
| Qualitative | 4† | 10.00 |
| Mix-method | 3† | 7.50 |
| Other | 10 | 17.54 |
| **Origin** |  |  |
| North America | 35 | 61.40 |
| Asia | 6 | 10.53 |
| Europe | 9 | 15.79 |
| Africa | 3 | 5.26 |
| Oceania | 2† | 5.00 |

*All studies (N=84) **Analysis strategy 1(N=71)

**S5 Table. Associated factors to increased perception of GD.**

| Associated factors to increased reported discrimination | Studies included |
| --- | --- |
| Both sexes: |  |
| Gender (women)* | [1]; [2]; [3]; [4]; [5]; [6]; [7]; [7] |
| Age (young)* | [8]; [6] |
| Higher education* | [9]; [10]; [11]; |
| Social class (I or II)* | [11][12] |
| Race* |  |
| White | [9] |
| Black | [4] |
| Minority | [6] |
| Individualism* | [13] |
| In women: |  |
| Neosexist believes (less)* | [14] |
| Age (young)* | [5] |
| Sexual harassment*† | [15]†[5]* |
| Reported sexual violence† | [16] |
| Higher education*† | [17]*; [11]**†** |
| Race |  |
| White † | [18][10] |
| Black † | [10] |
| Gender identity † | [19] |
| Social class (I or II)† | [11]; [20] |
| Gender related stressors† | [21] |
| Civil status (married or divorced)† | [11]; [20] |
| Specific work fields*† | [2]*; [15]**†**; [22]**†**; [23]**†;** [24]**†** |
| In men |  |
| Neosexist believes (more)* | [14] |
| Centrality of group membership* | [14] |

*studies performed both in men and women †studies performed only in women

**S6 Table. Reported associations to perception of discrimination**

| **Associations to GD** | **Studies included** |
| --- | --- |
| **Personal (n=16)** |  |
| Poor mental health*† | [8]*; [11]*; [25]*; [7]*; [26]†; [27]† |
| Poor self-reported health* | [28][12] |
| Lower health related quality of life† | [29] |
| Illicit drug use† | [30]; [26] |
| Lower self-concept |  |
| Low self-esteem† | [23]; [28]; [26]; [27] |
| Loss self-worth† | [31] |
| Low group-esteem† | [16] |
| Self-doubt† | [31] |
| Body-shame† | [32] |
| Self-objectification† | [32] |
| Feelings of distress† | [23]; [15]; [18] |
| **Professional (n=7)** |  |
| Low satisfaction† | [23]; [33]; [34] |
| Low self-concept† | [23]; [35] |
| Perception of stressful environment† | [23]; [22] |
| Career choices† | [18]; [36] |
| Burn out† | [34] |

*factors defined in women and in men †studies performed only in women

**S7Table. Studies used to induce the construct.**

| **Operative components** | **Studies** |
| --- | --- |
| **Undervaluation** |  |
| Recognition of performed activities | [23]†; [15]†; [37]†; [31]†; [35]†;[38]*‡;[9]*‡; [39]‡; [18]†; [33]†; [40]*‡; [41]*; [36]†; [42]†; [13]*; [43]†; [24]†; [44]* |
| Evaluation standards | [15]†; [35]†; [9]*‡; [39]‡; [33]†; [40]*‡; [36]†; [13]*; [43]†; [24]†; [44]* |
| Expectations of gender stereotype conformity | [45]†; [23]†; [15]†; [31]†; [35]†; [3]*; [38]*‡; [46]‡; [9]*‡; [39]‡; [18]†; [47]*; [48]‡; [33]†; [40]*‡ ¤; [41]*; [43]†; [44]* |
| Opportunities in access | [16]†; [45]†; [15]†;[37]†; [49]‡; [31]†; [35]†; [38]*‡; [9]*‡; [18]†; [47]*; [48]‡; [33]†; [40]*‡; [5]*; [41]*; [36]†; [42]†; [13]*; [17]*; [24]†; [44]* |
| **Different Treatment** | [49]*‡;* [9]**‡;* [39]*‡;*[18]*†;* [33]*†;* [40]**‡;* [50]**;* [44]**;* [27]*†* |
| Behavior | [3]**;* [46]*‡;* [9]**‡;* [40]**‡;* [44]**;* [27]*†* |
| Lack of Support | [31]†; [35]†; [38]*; [9]†; [18]†; [33]†; [50]*; [36]†;[42]†; [43]† |
| Less respected/ Disrespect | [31]†; [3]*; [46]; [9]**‡*; [39]*‡*; [21]†; [40]*‡; [36]†; [43]†; [24]†; [44]*; [27]† |
| Isolation | [23]†; [31]†; [35]†; [9]**‡*; [33]†; [40]; [50]*; [36]†; [43]†; [24]†; |
| Verbal abuse | [9]**‡;*[21]*†;* [43]†;[44]* |
| Derogatory comments | [15]†;[37]†; [49]‡; [31]†; [35]†; [38]*‡; [46]*‡;*[9]**‡*; [39]*‡*; [18]†; [48]*‡*; [33]†; [21]*†*; [36] †; [43]†; [44]*; [27]† |
| Incentives of gender role conformity | [15]†; [4]**‡*; [22]†; [51] †; [43]†; |
| Discouragement of non-classical gender role activities. behaviors or attitudes | [37]†; [35]†; [38]*‡; [18]†; [47]*; [52]‡; [4]**‡*; [9]**‡*; [43]† |

GD assessed in both genders*; GD in females†; other discriminations ‡

**S8 Table. Risk of Bias of studies used for construct definition.**

|  | Applicability of the item (NT=33) | Compliance | | Concordance^1^ |
| --- | --- | --- | --- | --- |
|  | NG (%) | N | % | % |
| **Qualitative study items JBI** | **(NG=4)** |  |  |  |
| 1 | 4(100) | 2¤ | 50 | 50 |
| 2 | 4(100) | 4* | 100 | 100 |
| 3 | 4(100) | 4* | 100 | 100 |
| 4 | 4(100) | 4* | 100 | 75 |
| 5 | 4(100) | 3* | 75 | 100 |
| 6 | 4(100) | 3 | 75 | 100 |
| 7 | 4(100) | 2 | 50 | 100 |
| 8 | 4(100) | 4* | 100 | 100 |
| **Cohort study items**  **JBI** | **(NG=1)** |  |  |  |
| 1 | 1(100) | 0 | 0 |  |
| 2 | 1(100) | 1 | 100 |  |
| 3 | 1(100) | 1* | 100 |  |
| 4 | 1(100) | 1 | 100 |  |
| 5 | 1(100) | 1 | 100 |  |
| 6 | 1(100) | 1* | 100 |  |
| 7 | 1(100) | 1* | 100 |  |
| 8 | 1(100) | 0 | 0 |  |
| 9 | 1(100) | 1 | 100 |  |
| 10 | 0(0) | - | - |  |
| 11 | 1(100) | 1 | 100 |  |
| **Cross-sectional items**  **JBI** | **(NG=26)** |  |  |  |
| 1 | 26(100) | 15 | 57.7 | 100 |
| 2 | 26(100) | 20*¤ | 76.9 | 88.5 |
| 3 | 18(69) | 16* | 88.9 | 100 |
| 4 | 17(65) | 7* | 41.2 | 100 |
| 5 | 13(50) | 12 | 92.3 | 100 |
| 6 | 13(50) | 11 | 84.6 | 100 |
| 7 | 21(80) | 12* | 57.1 | 100 |
| 8 | 26(100) | 26* | 100.0 | 100 |
| **Mix-method items**  (O’Cathain et al.. 2008). | **(NG=2)** |  |  |  |
| 1 | 2(100) | 1 | 50 | 100 |
| 2 | 0(0) |  |  |  |
| 3 | 2(100) | 2* | 100 | 50 |
| 4 | 1(50) | 1* | 100 | 100 |
| 5 | 1(50) | 1* | 100 | 100 |
| 6 | 2(100) | 1¤ | 50 | 50 |
| 7 | 2(100) | 2 | 100 | 100 |
| 8 | 2(100) | 1* | 50 |  |

NT. total N. NG: Group N.1Concordance was calculated dividing the number of items with the same answer to total number of items. Unclear categories were not considered lack of agreement *Unclear category was marked ¤items with no agreement included

References

1. Davidson MM, Gervais SJ, Canivez GL, Cole BP. A Psychometric Examination of the Interpersonal Sexual Objectification Scale Among College Men. 2013;60:239–50.

2. Pololi LH, Civian JT, Brennan RT, Dottolo AL, Krupat E. Experiencing the culture of academic medicine: Gender matters, a national study. J Gen Intern Med. 2013;28:201–7.

3. Stein M, Ph D, Oliver J. Gender Issues in Clinical Dental Education. 2014; March:401–10.

4. Inman ML, Baron RS. Influence of Prototypes on Perceptions of Prejudice. 1996;70:727–39.

5. Carr PL, Ash AS, Friedman RH, Szalacha L, Barnett RC, Palepu A, et al. Faculty perceptions of gender discrimination and sexual harassment in academic medicine. Ann Intern Med. 2000;132:889–96. http://www.ncbi.nlm.nih.gov/pubmed/10836916. Accessed 4 Nov 2017.

6. Ayalon L. Perceived Age, Gender, and Racial/Ethnic Discrimination in Europe: Results from the European Social Survey. Educ Gerontol. 2014;40:499–517.

7. Schmitt MT, Branscombe NR, Kobrynowicz D, Owen S. Perceiving Discrimination Against One’s Gender Group has Different Implications for Well-Being in Women and Men. Personal Soc Psychol Bull. 2002;28:197–210. doi:10.1177/0146167202282006.

8. Borrell C, Muntaner C, Gil-Gonz??lez D, Artazcoz L, Rodr??guez-Sanz M, Rohlfs I, et al. Perceived discrimination and health by gender, social class, and country of birth in a Southern European country. Prev Med (Baltim). 2010;50:86–92. doi:10.1016/j.ypmed.2009.10.016.

9. Puhl RM, Andreyeva T, Brownell KD. Perceptions of weight discrimination: Prevalence and comparison to race and gender discrimination in America. Int J Obes. 2008;32:992–1000.

10. Ro AE, Choi KH. Social status correlates of reporting gender discrimination and racial discrimination among racially diverse women. Women Heal. 2009;49:1–15.

11. Borrell C, Artazcoz L, Gil-González D, Pérez K, Pérez G, Vives-Cases C, et al. Determinants of perceived sexism and their role on the association of sexism with mental health. Women Heal. 2011;51:583–603.

12. Borrell C, Artazcoz L, Gil-González D, Pérez G, Rohlfs I, Pérez K. Perceived Sexism as a Health Determinant in Spain. J Women’s Heal. 2010;19:741–50.

13. Hang-yue N, Foley S, Loi R. The International Journal of Human Resource The effects of cultural types on perceptions of justice and gender inequity in the workplace The effects of cultural types on perceptions of justice and gender inequity in the workplace. 2007; December 2014:37–41.

14. Cameron JE. Social Identity , Modern Sexism , and Perceptions of Personal and Group Discrimination by Women and Men. 2002;45 December 2001:743–66.

15. Hinze SW, Journal I. ‘ Am I being over- sensitive ?’ Women ’ s experience of sexual harassment during medical training. 2004;:101–27.

16. Hagues R. “ The Girl Is Brought Up Knowing She ’ s Nothing ” : Listening to Voices of Tanzanian Women and Girls. 2018; August:109–17.

17. Yasukawa K, Nomura K. The Perception and Experience of Gender-Based Discrimination Related to Professional Advancement among Japanese Physicians. 2014;:35–42.

18. Settles IH, Pratt-hyatt JS, Buchanan NT. THROUGH THE LENS OF RACE : BLACK AND WHITE WOMEN ’ S PERCEPTIONS OF WOMANHOOD. 2008;32:454–68.

19. Wang K, Dovidio JF. Perceiving and Confronting Sexism : The Causal Role of Gender Identity Salience. 2016.

20. Borrell C, Artazcoz L, Gil-González D, Pérez G, Rohlfs I, Pérez K. Perceived Sexism as a Health Determinant in Spain. J Women’s Heal. 2010;19:741–50. doi:10.1089/jwh.2009.1594.

21. Berg SH. Everyday Sexism and Posttraumatic Stress Disorder in Women. Violence Against Women. 2006;12:970–88. doi:10.1177/1077801206293082.

22. Mccracken CM, Warner ET. Organizational Context and Female Faculty ’ s Perception. 2017;26:1–11.

23. Carr PL, Szalacha L, Barnett R, Caswell C, Inui T. A &quot;Ton of Feathers&quot;: Gender Discrimination in Academic Medical Careers and How to Manage It. J Women’s Heal. 2003;12:1009–18. doi:10.1089/154099903322643938.

24. Adesoye T, Mangurian C, Choo EK, Girgis C, Sabry-Elnaggar H, Linos E, et al. Perceived Discrimination Experienced by Physician Mothers and Desired Workplace Changes. JAMA Intern Med. 2017;177:1033. doi:10.1001/jamainternmed.2017.1394.

25. Jung-choi K, Jang S. Does the Experience of Discrimination Affect Health ? A Cross-Sectional Study of Korean Elders. 2016.

26. Kucharska J. Cumulative trauma, gender discrimination and mental health in women: mediating role of self-esteem. J Ment Heal. 2018;27:416–23. doi:10.1080/09638237.2017.1417548.

27. Kira IA, Hanaa S, Bujold-Bugeaud M. Gender Discrimination (GD): A Conceptual Trauma-Based Framework for GD and the Development of Gender Discrimination Inventory. Psychology. 2015;06:2041–70. doi:10.4236/psych.2015.616201.

28. Harnois CE, Bastos JL. Discrimination , Harassment , and Gendered Health Inequalities : Do Perceptions of Workplace Mistreatment Contribute to the Gender Gap in Self-reported Health ? 2018.

29. Logie CH, Wang Y, Lacombe-duncan A, Anne C, Kaida A, Conway T, et al. HIV-related stigma, racial discrimination, and gender discrimination: Pathways to physical and mental health-related quality of life among a national cohort of women living with HIV. Prev Med (Baltim). 2017. doi:10.1016/j.ypmed.2017.12.018.

30. Carliner H. Gender discrimination , educational attainment , and illicit drug use among U . S . women. Soc Psychiatry Psychiatr Epidemiol. 2017;52:279–89.

31. Babaria P, Bernheim S, Nunez-smith M. gender in health-professional education Gender and the pre-clinical experiences of female medical students : a taxonomy. 2011;:249–60.

32. Holland E, Koval P, Stratemeyer M, Thomson F, Haslam N. Sexual objectification in women ’ s daily lives : A smartphone ecological momentary assessment study. 2016.

33. Nye CD, Brummel BJ, Drasgow F. Differentiating Gender Discrimination and Sexist Behavior : An Examination of Antecedents and Outcomes. 2009;:299–314.

34. Moore LR, Ziegler C, Hessler A, Singhal D, LaFaver K. Burnout and Career Satisfaction in Women Neurologists in the United States. J Women’s Heal. 2019;28:515–25. doi:10.1089/jwh.2017.6888.

35. Robnett RD. Gender Bias in STEM Fields : Variation in Prevalence and Links to STEM Self-Concept. 2016;40:65–79.

36. Bruce AN, Battista A, Plankey MW, Johnson LB, Marshall MB. Perceptions of gender-based discrimination during surgical training and practice. Med Educ Online. 2015;20:25923. doi:10.3402/meo.v20.25923.

37. Derks B, Ellemers N, Laar C Van, Groot K De. Do sexist organizational cultures create the Queen Bee ? 2011;:519–35.

38. Cochran A, D M, Hauschild T, D M, Elder WB, S M, et al. Perceived gender-based barriers to careers in academic surgery. Am J Surg. 2013;206:263–8. doi:10.1016/j.amjsurg.2012.07.044.

39. Inequalities S, Discrimination P, Life M, Purpose H, Keyes CLM, Hughes DL. and Eudaimonic Status Inequalities , Perceived Discrimination , Well-being : Do the Challenges of Minority Life Hone Purpose and Growth ?*. 2015;44:275–91.

40. Gomez JP. Exploring cross-group discrimination: The subjective experience of inferiorizing events. 1998. https://deepblue.lib.umich.edu/handle/2027.42/130968. Accessed 22 Apr 2019.

41. Baqi S, Albalbeesi A, Iftikhar S, Baig-Ansari N, Alanazi M, Alanazi A. Perceptions of gender equality, work environment, support and social issues for women doctors at a university hospital in Riyadh, Kingdom of Saudi Arabia. PLoS One. 2017;12:e0186896. doi:10.1371/journal.pone.0186896.

42. Reid PT. Perceptions of sex discrimination among female university faculty and staff. Psychol Women Q. 1987;11:123–8.

43. Powell K. How female scientists can confront gender bias in the workplace. Nat 2018 5617723. 2018.

44. Miller J, Katz D. Gender Differences in Perception of Workplace Experience Among Anesthesiology Residents. J Educ Perioper Med JEPM. 2018;20:E618. http://www.ncbi.nlm.nih.gov/pubmed/29928665. Accessed 6 May 2019.

45. Heredia EB, Ramos A, Candela C. Laberinto de cristal en el liderazgo de las mujeres. 2011;23:173–9.

46. Chou T, Asnaani A, Hofmann SG. Perception of Racial Discrimination and Psychopathology Across Three U . S . Ethnic Minority Groups. 2012;18.

47. Miller K, Clark D, Miller K, Clark D. “ Knife before wife ”: an exploratory study of gender and the UK medical profession. 2008.

48. Zainiddinov H. Are all Muslims treated the same? Racial and ethnic differences in perceived discrimination among Muslim Americans. 2016. doi:10.7282/T3NG4STM.

49. Göçmen İ, Yılmaz V. Exploring Perceived Discrimination Among LGBT Individuals in Turkey in Education , Employment , and Health Care : Results of an Online Survey Individuals in Turkey in Education , Employment , and. 2016;8369 January 2017.

50. Mohd AZ, Ismail Z, Abdullah B, Daud S. European Journal of Obstetrics & Gynecology and Reproductive Biology Gender bias in training of medical students in obstetrics and gynaecology : a myth or reality ? Eur J Obstet Gynecol. 2015;186:17–21. doi:10.1016/j.ejogrb.2014.12.018.

51. Chisango T, Mayekiso T, Thomae M. The social nature of benevolent sexism and the antisocial nature of hostile sexism : Is benevolent sexism more likely to manifest in public contexts and hostile sexism in private contexts ? 2015;50:363–71.

52. Flippen CA, Parrado EA. Perceived discrimination among Latino immigrants in new destinations: The case of Durham, NC. Sociol Perspect. 2015;58:666–85. doi:10.1177/0731121415574397.

53. O’cathain A, Murphy E, Nicholl J. The Quality of Mixed Methods Studies in Health Services Research. J Health Serv Res Policy. 2008;13:92–8. doi:10.1258/jhsrp.2007.007074.
